# The synthetic biology puzzle: a qualitative study on public reflections towards a governance framework

**DOI:** 10.1007/s11693-015-9182-x

**Published:** 2015-10-05

**Authors:** Johannes Starkbaum, Matthias Braun, Peter Dabrock

**Affiliations:** 1 Department of Political Science, University of Vienna, Vienna, Austria; 2 Chair for Systematic Theology II (Ethics), Friedrich-Alexander-University Erlangen-Nuremberg, Kochstraße 6, 91504 Erlangen, Germany

**Keywords:** Emerging biotechnologies, Public engagement, Synthetic biology, Bioethics

## Abstract

Synthetic biology is currently one of the most debated emerging biotechnologies. The societal assessment of this technology is primarily based on contributions by scientists and policy makers, who focus mainly on technical challenges and possible risks. While public dialogue is given, it is yet rather limited. This study explores public debates concerning synthetic biology based on a focus group study with citizens from Austria and Germany and contextualises the analysed public views with content from policy reports and previous empirical studies on public engagement. The findings suggest that discussants favoured a gradual implementation process of synthetic biology, which is receptive to questions about the distribution of possible benefits. The discussed topics correspond in many ways with content from policy reports and former investigations, yet the emphasis of the discussions was different for many aspects.

## Introduction

Emerging biotechnologies operate at the threshold of technological innovation and public expectations. They have gained growing attention in the recent decades as scientists and policy maker likewise evaluate them as promising for different areas, including food and energy production, pharmaceutical industry or intellectual capital (Nuffield Council on Bioethics 2012; BBSRC and EPSRC 2011). One of the most frequently debated emerging biotechnologies is synthetic biology (SB). SB is widely understood as an umbrella term for a range of scientific practices bringing together scientists and engineers who either seek to develop novel bio-bricks, proto-cells or simple life-forms from scratch (bottom-up), or fundamentally modify existing organisms by implementing synthetic genes or proteins (top-down) (Singh 2014). The general objective is to generate and assemble functional modular components for the development of novel applications and processes within fields like agriculture, energy production and medicine. Beyond the first achievements within a synthetic version of the antimalarial compound Artemisinin (Carothers 2013) there are several SB-projects, which aim at contributing to the vaccine development for communicable diseases such as the Human Immunodeficiency—(Rerks-Ngarm et al. 2009; Hansen et al. 2013) or the Hepatitis C virus (Liang 2013). Beyond such scientific developments from the medical sector, SB is also supposed to offer novel opportunities for the creation of new industries with profound economic implications for the major economies (Klöck 2015).

Beside the envisioned benefits of SB, there are however several scientific, legal as well as ethical uncertainties. These are associated with the development of synthetic life, cells or genomes, and regarding their potential impact on the environment, biological diversity as well as human health (Scientific Committee on Health and Environmental Risks et al. 2015). Whereas some stakeholder rate the existing legal regulations (especially the EU Directives 2001/18 EC and 2009/41/EC) to sufficiently cover the current research action (Bar Yam et al. 2012; OECD 2014), other claim a need for an on going focus on biosafety and biosecurity issues (German Ethics Council 2014) as well as a focus on a possible lack of distinct laws for issues like ownership or trans-nationality (The US National Academy of Science 2013; BIOS 2011). There are furthermore technical and governance challenges that gain ongoing awareness, peculiarly concerning the possible ecological or health effects (Dana et al. 2012). Yet there is no clear vision of an appropriate governance frame regarding SB. Thus, one of the most urgent desiderata is to develop an innovative and sustainable governance frame in order to balance this new emerging biotechnology at a very early stage (BIOS 2011).

Nevertheless, there are several policy and governance recommendations on SB by different commissions and agencies published within the last 5 years. In general such recommendations advice to proceed research while making risks transparent (Nuffield Council on Bioethics 2012). In spite of all differences, three common topics can be identified amongst these reports. A frequently issue is (A) the question of a well-adjusted risk assessment. Regarding the aspect of bio-safety, questions on release, exchange with natural systems as well as unknown and possibly precarious future developments are discussed. Bio-security is most commonly related to intended misuse of bioscience for criminal intents or bio-terrorism (European Commission 2010; European Academies Science Advisory Council 2010; International Risk Governance Council 2010; Acatech 2012; Health and Security Executive 2012). Beside security questions the reports point out (B) the importance of intellectual and economic property issues. Thereby questions on the distribution of economic benefits (OECD 2010, 2014) or intellectual and legal ownership (European Group on Ethics 2009; US National Academy of Science 2013) are discussed. A third frequently discussed issue is (C) the question whether certain criteria must be met in order to call a governance strategy solid and responsible. The US National Academy of Science (2013), for example, published a summary report of an international symposium series that addressed, amongst other issues, regulatory challenges associated with the fluid boundaries, multiple applications and the yet unknown future of the field. Furthermore, the inclusiveness and the modes of engagement with various stakeholder are discussed. The inclusion and connection of stakeholder for sustainable governance includes, within most reports, to integrate the views and values of citizens and to therefore act in their interest while intensifying public dialogue (Presidential Commission for the Study of Bioethical Issues 2010). A report by BIOS (2011) attests the fragmentation of stakeholder and demands a stronger focus on cross-borderness. Thus, a common and sustainable governance strategy must integrate the views and values of different publics. It is therefore of utmost importance to foster and intensify the public-science dialogue.

Yet, including publics in scientific and technological innovation processes has always been controversial. In the 80es and early 90es of the last century, it was merely common sense that increasing information for (uninformed) publics will likely increase their acceptance of science (deficit paradigm). Different studies have however shown that publics develop different forms of expertise and that views of “experts” are only one valid viewpoint amongst others (Wynne 1996; Epstein 1996). Therefore, a new dialogue paradigm emerged that embraces concepts like engagement (House of Lords Select Committee on Science and Technology 2000), mutual inclusiveness (Prainsack 2014), and responsible research and innovation (Von Schomberg 2011). The latter is one leading principle for emerging biotechnologies in Europe which emphatically promotes an interactive and transparent implementation of technologies that likewise includes stakeholders, professionals and citizens (European Commission 2011; Von Schomberg 2011). Yet, including citizens in the governance of an emerging technology may occur in different ways, a common strategy within the European Horizon 2020 funding programme is to deliberatively engage (representatively polled or purposively selected) citizens in events, debates or interviews. Dependent on the methodology, different conceptions of “publics” lead these endeavours. While polls address publics foremost as all citizens within a political space (Law 2009), discursive qualitative methods like focus groups construct publics as temporary groups of interested or affected citizens (Dewey 2007).

Public engagement strategies in the field of emerging biotechnologies have not only been intensified during the last decades, Hansen and Metzler (2012) go as far as claiming the identification of a shift from a reactive to a prospective (anticipated) approach of public engagement within new technologies. Thus, different civil society organisations, stakeholder and institutions aim to generate public engagement at a very early stage of the innovation processes rather than to educate people for the sake of greater acceptance of technology (Acatech 2012). Whilst there exist numerous studies on public perceptions of different emerging biotechnologies, those that focus on SB are still few in number.

A Eurobarometer survey shows that representatively polled Europeans are divided in their attitudes towards the question whether the public should be actively involved in the governance of science and technology or if the provision of information is sufficient (European Commission 2013). The same Eurobarometer study shows that Europeans generally express a favourable approach towards science and technology. Nevertheless, most citizens say to have no knowledge about SB in particular (European Commission 2010; Pauwels 2009). The potential for public conflict with SB is thus still up for debate (Torgersen and Hampel 2012). Some scholars claim that most people have no interest in these technologies and that its potential for conflict is therefore overestimated. The special exhibition Gen-Welten (gene-worlds) that was launched in 1998 in four German-speaking cities, for example, gained much less interest and had much less visitors than expected (Schmidt 2001).

Many authors conclude that people make sense of SB based on former experiences with other biotechnologies (Kronberger et al. 2012; Torgersen and Schmidt 2013). While different empirical studies show that both, rejection and support, are given; supportive attitudes are mainly expressed concerning medical applications (BBSRC 2012; Kronberger et al. 2012; Pauwels 2009). However most stakeholder reason that citizens mainly take notice of personal risks rather than consumer benefits (Acatech 2012). This statement is backed up by empirical findings from another Eurobarometer study in which respondents expressed their wish for more knowledge of risks rather than of benefits (European Commission 2010).

However, a quantitative study conducted by the Hart Research Associates (2013) in the US reports a balanced perception of risks and benefits of SB by the public. The same study suggests that more information on SB might even fortitude critical attitudes. Once respondents were given more information, they tended to perceive risks more dominantly. However, in the ratio of two to one, Americans believe SB should be allowed to move forward rather than to be banned, which suggests a substantial support for this kind of research in the US. In detail, respondents expressed most concern about bio-warfare (28 %), the creation of life as moral rupture (27 %), possible negative health effects (20 %) and dangers for the environment (17 %). A related focus group study by the Hart Research Associates (2014) highlights once more that people perceive both, risks and benefits, while the latter are more dominant in conjunction with medical applications. During the discussions, the unforeseen and unintended consequences were the major issues rather than unintended usage like bio-warfare or bio-terrorism.

Being polled about concerns regarding SB, people from the EU-27 mostly emphasised the speed of change, unforeseen side effects and potential terrorist usage of new technologies from a list of different issues (European Commission 2013). A large qualitative and quantitative study by the German Institut für Demoskopie Allensbach and Leopoldina German National Academy of Sciences (Leopoldina 2015) stresses that Germans perceive SB as complex, distant issue, which is evaluated along perceived risks and benefits for particular fields of applications.

These studies about public perceptions of SB present a first glance unto this broad topic. We therefore set up a focus group study in order to explore and analyse how German and Austrian citizens perceive different applications that can be located at the gateway of Genetic Engineering and SB. Therefore, the study is interested in the reasoning patterns and to scrutinize public understandings of meaningful governance frameworks for this technology.

## Research agenda and methodology

The presupposition at the beginning of the investigation was constituted by the assumption that most people have not heard much details about SB. This presumption is also backed by a Eurobarometer study (European Commission 2010) that revealed that most Europeans said to have not heard of emerging biotechnologies so far. While any engagement with groups of citizens necessarily had to imply an introduction to the field of SB, we still aimed for an open and explorative approach.

On this basis, we conducted a focus group study in Erlangen and Nuremberg, Germany, and in Vienna, Austria to better understand how different groups of citizens perceive and reflect novel scientific practices. These locations were chosen as former investigations have shown that people in central European countries like Germany and Austria express comparably high concerns about emerging biotechnologies (European Commission 2010) and therefore provide a good sample for critical reflections.

Focus groups are discussions of small groups of people led by a professional moderator and are widely used in the field of research on public engagement (Bloor et al. 2001).They reveal how members of the public discuss and reflect certain issues addressed by a moderator. The major aim is to identify different understandings and shared patterns of meaning, rather than to produce representative data on certain populations. We sampled citizens for the respective focus groups and aimed for homogeneity in terms of the participants’ age and education, whereas the overall sample provided a range of people with multiple socio-demographic backgrounds. The individual groups therefore consisted either of younger (18–35 years) or mature/older people (35+ years), and respectively either of people with higher or lower educational level. While many of our discussants had a high educational status (tertiary education), groups with people of lower education levels participated as well. Each discussion group consisted of about six to eleven people. The sample of all nine groups together consisted of 37 women and 32 men, whereas the average age was 39. We recruited a balanced sample of people with different employment statuses. All individuals were recruited via online platforms and multiplicator persons (snowballing), totalling 69 participants. Each group session lasted for about one and a half hour (Table 1).

**Table 1:** Demographics of focus group participants

	Total
Focusgroups	
Total	9
Participants	
Total	69
Female	37
Male	32
Age	
Range	18–76
Mean average age	39
Highest (current) education level	
0–1 first stage basic education	4
2 lower secondary or second stage of basic education	6
3 upper secondary education	18
4 post-secondary non-tertiary education	6
5–6 tertiary education	35
Employment status	
Employed or freelance	25
Retired	18
In education	18
Unemployed or unpaid work	8

The discussions were arranged with an open approach, but with alertness to provide a reliable data set. This means that all groups followed the same topic guide applied by the same moderator. Participants were introduced to the field of emerging biotechnologies with a focus on genetic engineering and SB. It was explained that all organisms rely to some extend on a construction plan consisting of DNA, and that this plan sets a frame for its appearance and abilities. It was then explained that humans have interfered with this construction plan for several hundred years by breeding animals or specific plant types. Novel scientific practices have provided the opportunity to directly change or alter existing DNA structures and are therefore capable to achieve different effects. All groups were then introduced stepwise to three different areas of application, synthetic (1) fungus, (2) viruses and (3) algae with anticipated applications in the respective fields of (1) drug-development, (2) agriculture and (3) bio-fuel.

Case (1) is a widely cited example of the synthetically reproduced active substance Artemisinin that is the vital ingredient of lots of anti-malaria-drugs (Ro et al. 2006; Van Noorden 2010; Westfall et al. 2012). During the session, focus group participants were explained that a yeast fungus was genetically modified so that it started to produce Artemisinin such as the Artemisia Annua plant, and that this synthetic substance was then reprocessed to anti-malaria-drugs for clinical trials.

In case (2), participants were told how scientists experimented in order to synthetically produce viruses with the aim to affect a certain type of insect, which, from an agricultural perspective, is considered as being a vermin. Furthermore, it was claimed that it is anticipated that this virus will not affect other animals and that it will not be subject to evolution. Scientists would follow several approaches using viruses for insect control in agriculture, ranging from sterilisation to directed mutations (Fu et al. 2007; Son et al. 2006; Szewczyk et al. 2006).

The last case (3) addressed the use of algae-based bio-fuel production. It was communicated that algae is seen as a possible alternative to fossil fuels and that scientists aim to optimise the efficiency by altering the genetic structure of algae. Strategies include creating plants that depend on less light or making them produce more oil for refinement (Service 2011).

In each case we communicated that although the stressed applications and security measures are anticipated aims by scientists, it is yet not clear whether these aims might be achieved or if security measures will take full effect. Regarding each case the discussants were asked about their thoughts on these specific applications and which societal and governmental implications they assumed. The groups were given about 20 min per case to freely discuss their views. At the end of the discussions, the participants were asked about the similarities and differences between the three cases and how the presented technologies should be governed.

All data was audio-recorded and then transcribed for further analysis. The study’s aim was furthermore to go beyond the manifest content of the participants’ statements and revealing latent structures of meaning (Smithson 2000). We therefore conducted a theoretical coding as described in contemporary Straussian approaches of interpretive research and grounded theory (Clarke 2005; Charmaz 2006). A coding structure was created with the technical support of a qualitative data analysis software. Categories and codes were set both inductively (from the empirical material) and deductively (from prior studies and the topic guide) and were amended with commentarial information in order to reach higher reliability and data-richness. The main categories addressed (1) the perceived implications of emerging biotechnologies, (2) the strategies and expectations expressed by the participants, (3) the group dynamics and discursive elements (such as approval, rejection, speech flows, etc.) and finally (4) the different cases the participants listed and referred to in a content-related way. As concluding steps, the relation between the different cases and categories were explored in depth. The findings were summarised and prepared in memos that were adjusted along the whole process of the analysis. The following chapter provides an overview on the most relevant findings.

## Results

### Benefits

During the introduction to each case by the moderator, discussants were explained what aims scientists strive for with certain technologies such as (1) developments of malaria drugs, (2) reduction of insect populations and (3) bio-fuel. Overall, discussants initially expressed limited knowledge on these cases and therefore debated on the basis of the given information as well as former experiences they collected from other fields such as science and media. Besides critical attitudes, the discussants also randomly referred to these possible benefits of SB. Benefits for medical applications were comparably far more often addressed than the others. 45 sections where discussants referred to drug applications were coded, in comparison to 16 in the field of bio-fuel and only six within the field insect population control.

The participants described the necessity for novel and widely accessible anti-malaria drugs, sustainable bio-fuels from renewable resources and the need for optimising the food production. It was mostly debated how and whether these new approaches and techniques would lead to the anticipated aims. Those groups whose members had higher educational levels referred much more often to benefits of research in comparison to those people with a lower educational level. With the latter, drawbacks were given higher priority in the discussions.

However, with all groups, benefits were almost always negotiated along with anticipated risks and ‘negative implications’. These imagined drawbacks often included wide impacts such as environmental hazards or noxious side effects. The participants also referred to the usage of certain practices, which they considered as being morally wrong, as for instance the unavailability of drugs for certain population groups or the monopolisation of financial gains. A general understanding of the necessity of research was however given during the group discussions, albeit being paired with a critical stance and an awareness for alternative approaches.

In particular with the topics synthetic algae (coded 33 times) and synthetic viruses (coded 17 times), people questioned the necessity of SB for achieving certain benefits. Solely when people referred to drug applications this discourse was not prevalent (coded 6 times). The groups typically found consensus on a certain problem like increasing fuel demands but at the same time questioned whether high risk technologies were the preferable solution. On account of this, people discussed how renewable energy sources, mixed crop cultivation or abstemious lifestyles might be better solutions.

The following example 1 demonstrates how groups typically negotiated benefits and associated risks.

Example 1: Erlangen, 18–35 years old, higher education level

T4: Malaria is indeed a huge problem in Africa and South-Asia (…) therefore I find this research very important, essential (…). You cannot discuss it ethically because there are no embryonic stem cells in use.

T1: This sounds promising, however, I would be careful to legitimate this kind of research with solutions to social and medical issues in developing countries (…) because I do not see the problem with the amount of malaria drugs (…), but with the distribution.

T7: But how do you want to fight malaria? I mean you have to develop a drug (…).

T1: True, I would agree but I think there are already enough drugs for malaria. The problem is that those people who really need them often lack access due to patents, due to economic dependencies.

T8: Of course not, but with a delay of the typical 20 years indeed. This is of course a long time (…), but the hope is that it helps those people in 20 years.

T3: Maybe! For me it matters how these drugs get used (…). It would be a pity if it, again, would be intended for Europeans only.

T5: I cannot imagine that those who do research on this drug think about helping the 3rd World. I think that they do research for earning money.

The selected unit starts with the framing of malaria as a serious problem in Southern parts of the world and the conclusion that research should be conducted. It is followed by a justification that this sort of research is ethically valid in comparison to other approaches like stem cell research. The next argument stresses suspiciousness and the reassessing of the necessity of this approach (merely a distribution issue). Right afterwards benefits (medication) are brought up again as a supportive argument for this sort of research. The response condenses the argument of distribution to questions of access and monopolisation. Another respondent acknowledges the criticism but directly addresses the long term redemption. The statement is followed by another argument that criticises the uneven global distribution. The unit then closes with questioning the integrity of those people who conduct research and their intentions for the common good.

This first example shows how single topics were interwoven and negotiated along other issues. Questions of benefits were strongly linked to distribution and its governance. The next section will address these questions in more detail.

### Distribution

Discourse addressing commercial interests and monopolies was randomly present during the focus group sessions. Most groups found consensus on the issue that the output of this sort of research promise great economic value and therefore wondered about how gains and benefits were distributed. The participants debated the role of corporations and Western societies and how these entities capitalise power and resources. Many discussants expressed the feeling that there is invisible correlation between having the lead with science and its research programs and results of research and an economic reasoning, which is embedded as an important but in transparent driver of research.

The dominant discourse on distribution was not directed towards rejecting biotechnologies, but towards creating the awareness of doing things “the right way”. The respective examples used were either companies or corporations that capitalise financial gains from drug or fuel production, or the “West” taking advantage of the “Global South”.

Even though the subject matter of just distribution was indeed a topic of great relevance to all groups, there were large differences between the different areas of SB-application. Codes that address distribution, monopolisation, or patenting issues were coded 53 times with the case of synthetic fungus/drug, and comparably much less frequently with algae/bio-fuel (23 times) or virus/agriculture (12 times). However, it must be considered, that this imbalance was also triggered by the usage of a case that included malaria, a disease, which typically occurs in Southern countries and therefore enhanced discussions about global justice.

Another noticeable difference was demonstrated by the educational level of the groups. Whereas those groups with discussants with higher education reflected more on global distributional justice, fairness and monopolisation in general, the lower-educated groups did oppose economic interests of corporations more often. The groups with higher education typically prone to ask if certain groups (e.g. Global South) were excluded from research benefits.

The strong discourse on a democratisation of risks and benefits showed how research and its products were associated with wider consequences and impacts.

### Systemic and process-oriented understandings

Regardless if the groups discussed benefits, distribution or other related risks, a sensitiveness to interrelations and consequences could be observed. It could be seen that risks were in many cases not directly addressed in an essentialist way towards the products of science itself (such as a synthetic drug or synthetic virus) but rather to its anticipated impacts and indirect implications on existing systems. Influencing or controlling possible impacts was mostly debated on the level of expert-regulation and technical or legal solutions.

In the case of synthetic fungus/drug, the participants discussed how a synthetic drug may have (no) implications on Southern parts of the world or unknown long-term implications on patients. Both issues address indirect consequences and wider impacts. However, within this example, the participants agreed that all options for controlling science and its products via tests and clinical trials should be exploited. Possibilities for control were mainly anticipated by those groups with higher education, whereas those groups with lower education did only seldom debate possibilities for control and containment. With both other examples, and in particular with the case of virus/agriculture, the discussants were less optimistic about possibilities for control.

The case of algae/bio-fuel typically triggered a discourse on environmental impacts and the disruptions of system balances. Likewise to the first case, the groups seldom associated hazards with a synthetically created alga itself, but with the wider impacts it might have. Subsequently, in the participants’ view its release might disturb the natural system, which is considered as being in balance. The actual imagined impacts ranged from the replacement of other species of algae to environmental hazards or indirect impacts on humans if algae find their way into the food chain—either as a dish or via seafood that was exposed to it. In addition it was assumed that a synthetic alga may be subject to evolutionary processes. The prevalent option of containment was considered as a containment of algae in closed tanks.

The case of virus/agriculture was debated with the highest level of anxiety. Concerns about the unleashing and irreversible consequences were dominant during the discussions. These concerns were even strengthened by the shared understanding that a virus may always mutate, even if designed to stay stable. People thereby referred to examples from the media, such as movies on epidemics, as well as to knowledge about flu viruses that alter each year. The anticipated impacts ranged from invasive effects on humans to environmental hazards and disruptions of insect populations. All groups spotted limited options for controlling or containing a synthetically created virus.

The products of emerging biotechnologies were discursively processed in a systemic understanding of nature, science and technology, rather than on the base of their ontological status. The groups thereby followed a causal rather than an essentialist approach. This means that the causes and effects, and in particular the indirect ones, gained most attention in the groups’ discussions, rather than the objects themselves in their constitution and existence. Special unease could be noted regarding the idea that the hybrid objects of science get unleashed and embedded into the world. The following example 2 demonstrates how the case of a virus triggered particular concerns in relation to a drug based on synthetic ingredients.

Example 2, Vienna, 18–35 years old, lower educational level

T1: You cannot make tests in the outside world. You cannot say how warm it is, how much wind there is, how fast it will spread.

T3: I understand, (…) there are other conditions out there, but I do not think that they are fully controllable.

T5: But I think that drugs, I mean, there is a human attached, the consumer. (…) but what is outside cannot be influenced completely, cannot be controlled. And yes, the drug is by then finished, a finished product.

T2: If the drug is harmful to the human body, you can withdraw it, yes? But if I unleash a virus out in the nature, what can I do then? I cannot tell him, “come back to the lab, I made a mistake”. It is then out there and reproduces and spreads. You must activate instruments against this, but those are possibly not applicable. I think about a movie called ‘the deadly virus’.

T5: Because a virus can alter.

T2: Right, it mutates and mutates on and they have found a cure, but it is the wrong one, and, humans die like flies [laughing]. If I imagine it in a corn field and I consume bread and get something, what do I know? And doctors have only limited knowledge; they do not know what I am ill from (…). A virus is too unpredictable.

T6: Yes, as long as diseases like HIV are not completely explored, one should be careful with a virus. Because HIV is an example that viruses are unpredictable.

The section starts with claiming that tests cannot be made in the outside world because the conditions are not stable. This thought is followed by the perception that drugs cannot be controlled. T5 in turn stresses that a drug is much closer in relation to a virus that is “out there”. This leads to the conclusion that the latter cannot be controlled, whereas a drug is an isolated product. This argumentation supports the first statement. Another participant jumps on the bandwagon and agrees that a drug might be withdrawn from the market, whereas an unleashed virus cannot. This statement is backed up by some media content. T5 sharpens the discourse by referring to the possibility of alteration—T2 directly supports this argumentation and continues to link it with media examples. This leads to the conclusion that even experts may not be able to help and understand the issue, as it is unpredictable. The last argument refers to the lack of fully understanding HIV.

This short example shows how a group of people finds a consensus about a synthetic virus for agricultural purposes and labels it as being uncontrollable. The described discussion represents a typical example of the limited divergent opinions within this specific discourse and displays that this perception was shared among the discussants. However, even though this example indicated serious concerns, there were still benefits expected concerning SB that were balanced with the anticipated risks during the discussion.

## Discussion

The provided focus group data reveal detailed insights into public discourses on SB and related preferences for a governance framework. On a very general level, discussants emphasised wide-ranging complexity in this field while reflecting on the implementation of products from science into society and environment. As far as aims of research were appreciated, a gradual approach receptive to questions of distribution was predominant. The ways in which scientists aim to achieve benefits were challenged by the discussants on a regular basis: discussants not only debated possible risks but considered also alternative solutions for achieving certain research aims. The observed public views revealed both, parallels and differences to the discourse from policy reports as well as to prior studies in the field of public engagement.

Yet, we extracted five key aspects for a governance framework that is respective for citizen’s views. These aspects constitute the intersection of the presented results and the content of the described policy reports and studies: (a) differentiation versus generalisation, (b) technical challenges and benefits, (c) bio-safety and bio-security, (d) distribution, patenting and ownership, and (e) public engagement:

(a) *Differentiation versus generalisation* The assumed or envisioned area of application made a difference for the way people evaluated SB. We have shown—as other studies on public views did (BBSRC 2012; Kronberger et al. 2012; Pauwels 2009)—that support of SB is mostly expressed within the field of medical applications in comparison with other fields. Policy reports only seldom distinguish between areas of application within the large field of SB and mostly provide valuations and recommendations for governance on a general level. This shows that it might be worthy to discuss differentiations more often. It was not only the general support of an emerging biotechnology that varied in its intensity, but also the different approaches to address certain topics. Whether discussants spoke about distributional issues, benefits or risks depended on which of the three cases was addressed. The case of malaria, for instance, triggered intensive debates about distribution and fairness, whereas synthetic viruses revealed concerns about an assumed incalculability.

The age of the discussants showed almost no impact on the way people discussed issues of SB. Comparatively, the educational background showed indeed impact on the way of reasoning and the topics which were set at stake. Those groups with higher educational status named anticipated benefits more often and perceived options for regulating SB more likely than those groups with lower educational levels.

(b) *Technical challenges and benefits* The discussants have been confronted with anticipated benefits for each case: a malaria drug, insect population control and bio-fuel. We have reported a general acknowledgement of these aims, but also a critical reflection of the methods of production. The techniques of SB were challenged on a regular basis during the discussions, and alternative approaches were contemplated. A typical example was to give preference to electrically powered cars over the creation of bio-fuel. Other than in policy reports, discussants did—due to their lacking expertise—not discuss technical details, but showed awareness about the complexity and risks of this SB-technology.

Complementary with previous studies in the field of public engagement (Hart Research Associates 2013; European Commission 2010) we have demonstrated that citizens show either a balanced perception of risks and benefits, or that they discuss risks as prevalent. At the same time, previous studies suggest that in general most citizens approve research with SB. The empirical data presented in this article supports the observation in so far, that discussants perceived both, risks and benefits. During focus group discussions it became even evident that benefits and risks could not be separated as they were always debated in interrelation. As discussed in the first aspect (a), the relation of risks and benefits is not stable among time and cases—which calls for a differentiated governance approach.

(c) *Bio-safety and bio-security* Questions of bio-safety and bio-security are a dominant topic in policy reports on SB. Possible accidental or purposive impacts of synthetic entities on humans, humanity or the environment are with no doubt one major point of attention. This manifests also in the EU-Directives that typically come up in the context of SB, as shown in the introduction. Previous research on public engagement showed comparable findings. During the surveys conducted by the European Commission (2013) and the Hart Research Associates (2013), respondents most often chose items from the field of bio-safety and bio-security as most prominent. Qualitative focus groups data from the Hart Research Associates (2014) however, found more discussion on unforeseen and unintended consequences, rather than on bio-security issues. This corresponds with our data.

The presented focus group data revealed that bio-safety issues were comparably far more often topic of discussion than bio-security issues. The data furthermore showed how the linking and embedding of SB mattered more than their ontological status. There were only limited reflections on the rupturing of binary categories such as living and non-living or nature and culture (Braun et al. 2013). People focussed more on the processes triggered by and the embedding of synthetic entities as well as its impacts on humans, society and the environment. Our discussants shared the perception of a wide-ranging complexity and multiple interests in this field, such as by the industry or single states. The unleashing of synthetic entities were associated with chains of reactions that are hardly predictable. Contrasting policy reports only few reflections on biological warfare or biosafety were found in our focus group discussions. The issue only came up during the debate on virus/agriculture. The groups typically switched to other topics soon once the topics of biological warfare and bios security were brought up, which indicates that other issues were more important to them. Yet, it might also result from limited information on this aspect. Either way, the presented data suggests that citizens’ mainly desire realistic scenarios about the implementation of SB in society and the environment, as well about the involved actors.

(d) *Distribution, patenting and ownership* Reflections on patenting and ownership are dominant across policy reports on SB. They refer to questions about legal ownership of products from SB and findings of respective research. This includes also the given possibilities to patent designed products. The distribution of benefits among various stakeholder is seldom addressed in reports. Likewise, previous studies on public perceptions show limited data on these issues.

Within our focus groups, discussions about the distribution of benefits were dominant over questions of legal ownership. This was particularly visible with the case of synthetic drugs, which triggered debates about global justice. Predominantly private companies from the pharmaceutical industry were believed to operate in their own interest. Nevertheless, the involvement of private companies was also valued as necessary for the sake of financing and the production of applicable products. This reveals a different focus than those, which can be found in policy reports. Whereas legal ownership might be of utter importance for other stakeholder, such as industry partners or legal scholars, citizens showed less interest in this issue.

(e) *Public engagement*: The focus group discussants mostly expressed limited knowledge about biotechnologies and SB but did not raise their own information deficit as a problem. In fact, having limited knowledge was randomly constructed as group consensus. While these findings match the results from the Eurobarometer (2013) on the dichotomy between public education and engagement for the EU-27, there were still informed debates going on. Discussants randomly referred to the given information about SB, experiences with other technologies and cases from media. This way, other forms of expertise and knowledge were constructed during the debates.

Yet, options for control and containment of emerging biotechnologies were almost only trusted when performed by external experts or legal entities, rather than through individual action or personal competence. If influencing technological developments was perceived as possible, it was expected that the field was either regulated by legal and technical frameworks or by experts, such as policy makers or scientists. Bottom-up strategies, such as public protest, were almost never mentioned by the groups. The tenor was a somehow passive approach that may result from the expansive character of emerging biotechnologies and the thus perceived great level of complexity. The group situation might have also influenced this approach as respondents might have held back ideas about bottom up initiatives to avoid criticism by other group members if these control options were seen as unrealistic.

In summary, the study’s participants debated the provision of a leap of faith to science paired with a regular option for emergency exits: a granted loan. This governance modes acknowledge the anticipated aims of research but includes elements of (public) observation. A granted loan is a modus initiated in response to the perception that SB is still in the making and its (possibly irreversible) impacts are yet still unclear. Although people might approve scientific progress because of the expected benefits, they are also aware that research is scheduled to run long-term and that its impacts may go far further. Citizens perceive to have little influence on this development and thus demand technical, legal and political control mechanisms that may interfere at anytime necessary. In practice this implementation of granted loans is indeed a challenge.

It is a well-known dilemma that scientists may depend on long-term consent for research purposes while they cannot precisely predict the future benefits and risks of emerging biotechnologies. Innovation does, however, always imply a certain level of risk—and most discussants were well fond of this. Therefore, SB was mostly appreciated in principle, but scientific proceeding in the field was expected to happen in gradual approaches. During a symposium on SB Iain Gillespie of the OECD stated that “it is important to create governance systems which are sustainable, forward-looking and dynamic and which allow innovation (…) to emerge” (OECD 2010, 35). Correspondingly, the focus group discussants indeed stressed that security measures may not be integrated at any price, and must certainly account for innovation, too. Approaches are to be found, which implement flexible elements in new (technical) solutions that address the aimed-for social challenges. At the same time, efforts for democratising risks and benefits are to be strengthened on a global level and among experts, corporations and the broader public.

While stakeholder reports indeed stress comparable issues like arranged groups of citizens, this piece of research indicates that many details in reports and focus group discussions do differ. Public engagement is anymore mandatory for the growing and altering field of emerging biotechnologies. Particularly regarding SB the up-streaming activities are yet overall underrepresented.
